# Effects of physical activity and use of digital devices on visual acuity in children and adolescents during the COVID-19 pandemic: A cross-sectional study

**DOI:** 10.3389/fpubh.2022.1017479

**Published:** 2022-12-08

**Authors:** Xiao Zheng, Lei Shi, Weiyan Ou, Yaqing Xue, Ying Xu, Benli Xue, Jiachi Zhang, Pengyan Liang, Wei Huang, Zuguo Qin, Chichen Zhang

**Affiliations:** ^1^Department of Health Management, Shunde Hospital, Southern Medical University (The First People's Hospital of Shunde, Foshan), Foshan, China; ^2^School of Health Management, Southern Medical University, Guangzhou, China; ^3^Division of Physical Education, Hygiene and Arts Education Department of Education of Guangdong Province, Guangzhou, China; ^4^Health Publicity and Education Center of Guangdong Province, Guangzhou, China

**Keywords:** poor visual acuity, children and adolescents, COVID-19, physical activity, digital devices, health management

## Abstract

**Purpose:**

To determine the association between poor visual acuity, the use of digital devices and physical activity (PA) during the COVID-19 pandemic.

**Methods:**

A total of 327,646 Chinese children and adolescents were included in the analysis using a cluster random sampling method; this is a case-control study, of those 144,708 children and adolescents with poor visual acuity were included in the case group, while 182,938 who did not have poor visual acuity were included in the control group. A logistic regression model was used to assess the contribution of PA and the use of digital devices to poor visual acuity.

**Results:**

A total of 144,708 children and adolescents experienced poor visual acuity during the COVID-19 pandemic; 54.8% were male, and 55.2% live in rural areas. Compared to controls, children and adolescents with poor visual acuity exhibited more time for the use of digital devices (4.51 ± 2.44 vs. 3.79 ± 2.34 for cases and controls, respectively; *P* < 0.001) and PA (3.07 ± 0.92 vs. 2.85 ± 1.00 for cases and controls, respectively; *P* < 0.001). During the COVID-19 pandemic, risk factors related to poor visual acuity among children and adolescents included the use of digital devices (OR 1.135; 95% CI 1.132–1.139), and PA (OR 1.269; 95%CI 1.259–1.278). The results of interaction analysis show that for children and adolescents aged 12 to 17, the positive association between the use of digital devices and poor visual acuity decreased. The interaction effect between PA and digital devices is 0.987.

**Conclusions:**

Children and adolescents were at risk of poor visual acuity during the COVID-19 pandemic. Extended use of the digital devices increased the risk of poor visual acuity, especially for children aged 6–11 years. But the risk of poor visual acuity among children and adolescents decreases as the time spent on PA increases.

## Background

Myopia has emerged as a major health concern worldwide, particularly in East Asia (1). In June 2020, China Ministry of Education conducted a survey on the visual acuity of 14,532 students from primary, middle, and high schools. The results showed that compared with the data at the end of 2019, the myopia rate of students increased by 11.7% after the COVID-19 outbreak (2). In response to the COVID-19 outbreak, many countries adopted a series of control strategies (3). These measures significantly reduced the number of cases, including the closure of schools, home quarantine, and social distancing (4). According to the United Nations Educational, Scientific and Cultural Organization (UNESCO), more than 160 countries implemented nationwide closures, affecting over 87% of students worldwide (5). A nationwide school closure was implemented as an emergency measure to prevent the spread of COVID-19 among children and adolescents in China (6). China Ministry of Education estimated that more than 220 million children and adolescents are confined to their homes. Therefore, online courses were offered in a well-organized manner to ensure continuity of school learning and improve students' educational attainment (7). Online courses for primary and secondary schools are being offered from February 2020, lasting 3–4 months (8).

Recent research has mainly focused on unhealthy behaviors caused by the closure of schools, such as fewer outdoor activities, longer use of digital devices, irregular sleep patterns, and unhealthy diets among children and adolescents during the COVID-19 pandemic, as well as the negative effects of such behaviors on physical and mental health (9–11). The impact of reduced physical activity (PA), decreased outdoor time, and increased use of digital devices on visual acuity caused by quarantine measures worldwide has been largely ignored. Liu et al. (9) suggested that with the implementation of control measures, such as school closure, children and adolescents were physically less active and used digital devices for longer, periods, exhibited irregular sleep patterns, and adopted unhealthy diets, resulting in weight gain and loss of cardiorespiratory fitness. Pellegrini et al. (12) identified an increased risk of myopia after home quarantine. Wong et al. (4) reviewed studies on the associations between the use of digital devices, near work, outdoor time, and myopia, presenting the risk impact of increased use of digital devices on myopia during the COVID-19 pandemic. Although the etiology of myopia remains unclarified, education as one of the environmental factors has been correlated with them (13, 14).

The high prevalence of myopia among children and adolescents was found to be attributable to several factors, including the level of education, time spent outdoors, PA, and use of digital devices (15–18). According to some research, myopia was shown to be more common in students who studied for more than 5 h each day, and they think myopia is substantially more common in Singapore, Korea, and China than in other nations, presumably due to the high-pressure education systems (19). Rose et al. (20) found that “higher levels of total time spent outdoors, rather than sports *per se*, were associated with less myopia.” He et al. (21) pointed out that increased outdoor activities at school contributed to a reduced incidence of myopia among school children. Children and adolescents were not allowed to go outdoors, and could only exercise indoors due to COVID-19 containment measures. Some scholars have proposed the potential impact of home quarantine and online courses on myopia among children and adolescents (22). However, few large-scale empirical study has yet been conducted to validate this proposition. This study aimed to assess the impact of PA and the use of digital devices on the visual acuity of children and adolescents during the COVID-19 pandemic.

## Methods

### Sample

A stratified cluster random sampling method was used to collect data between May 8 and June 30, 2020. To build our sample, 5% of primary and secondary schools from each city in Guangdong province (21 cities) were randomly selected using equal probability method. Each city's education department has a list containing all schools in the region. The schools included in this study were selected by the education department based on this list, using the random number table method. A cluster sampling method was used to extract students from these schools, and the probability of each student being selected was the same. In this study, children and adolescents reported visual acuity in 2019 and 2020, respectively. We screened the sample based on the visual acuity of children and adolescents in 2019. Inclusion criteria: Children and adolescents aged 6–17 years; Without poor visual acuity in 2019. Visual acuity of children and adolescents in 2020 was analyzed as an outcome variable.

Respondents in the target population completed the Chinese version of the electronic questionnaire through an online survey platform (SurveyStar; Changsha Ranxing Science and Technology). The survey link was sent to the cell phone of the child's guardian, and guardians were asked to provide consent before the child could participate.

This is a case-control study. The questionnaires were anonymized to ensure data confidentiality and reliability. There were 356,552 children and adolescents were included in this study. Questionnaires in which the visual acuity status was left unfilled were excluded. Finally, 327,646 valid questionnaires were returned, with a response rate of 91.9%. Visual acuity of children and adolescents in 2020 was analyzed as an outcome variable. In this study, we defined children and adolescents with poor visual acuity were case group, and people without poor visual acuity were control group.

### Measurements

#### Dependent variables

The dependent variables included daily time spent on digital devices and PA during the COVID-19 pandemic. Demographic variables included sex (male, female), age (6–17), and residence (rural, urban).

#### Outcome variable: Poor visual acuity

In China, the Ministry of Education introduced “Administrative Measures for the Health Examination of Primary and Secondary School Students” in 2008 as part of a health surveillance program (23). Students of all grades were asked to perform a visual acuity test. The logarithm of the minimum angle of resolution (LogMAR) chart by ophthalmologists was used (24). LogMAR (using the Standard for Logarithmic Visual Acuity Charts, GB/T 11533-2011 of the Standardization Administration of the People's Republic of China) is the “gold standard” used by majority of clinical trials or interventions (25, 26). Poor visual acuity was defined as a UCDVA (LogMAR) < 5.0.

#### Analyses

The differences between cases and controls were compared using the two-sample student's *t*-test and chi-squared test. First, a logistic regression model was used to assess the contribution of PA (h/day) and the use of digital devices (h/day) to poor visual acuity. The outcome measure was poor visual acuity. The model was adjusted for age, sex, and residence. Second, we tested the two-way interaction of digital devices and age or PA (age × use time of digital devices; PA × use time of digital devices) on poor visual acuity among children and adolescents. Finally, we analyzed the effects of digital devices on poor visual acuity of children and adolescents at different ages using marginal effects. All analyses were performed using Stata software (version 15.0). All reported *P*-values were 2-sided.

## Results

### Difference between cases and controls

In 2020, A total of 144,708 children and adolescents experienced poor visual acuity were included in case control. Of which, 79,343 (54.8%) were male, most respondents were aged 8–12 years (96,143, 66.4%), and average age was 10.56, and 79,886 (55.2%) lived in urban areas. A total of 182,938 children and adolescents were included in the control group, the average age of respondents was 10.29, 95,095 (52.0%) were male, and 107,038 (58.5%) lived in urban areas. There were differences between cases and controls according to sex, age, and residence (*P* < 0.001). There were differences between cases and controls in the time of PA, but the difference was small (3.07 ± 0.92 vs. 2.85 ± 1.00 for cases and controls, *P* < 0.001). Children and adolescents with poor visual acuity spent more time on digital devices than controls (4.51 ± 2.44 vs. 3.79 ± 2.34 for cases and controls; Table 1).

**Table 1:** The poor visual acuity of children and adolescents during the COVID-19 pandemic.

**Variables**	**Case group**	**Control group**	***X** **^2^/t***	***P***
**Sex**			263.176	< 0.001
Male	79,343 (54.8)	95,095 (52.0)		
Female	65,365 (45.2)	87,843 (48.0)		
**Age (year)**			1,540.673	< 0.001
6	1,100 (0.7)	1,991 (1.1)		
7	15,360 (10.6)	24,845 (13.6)		
8	21,744 (15.0)	31,232 (17.1)		
9	18,267 (12.6)	23,912 (13.1)		
10	19,208 (13.3)	23,537 (12.9)		
11	18,399 (12.7)	20,713 (11.3)		
12	18,525 (12.8)	20,252 (11.1)		
13	11,698 (8.1)	12,550 (6.9)		
14	8,279 (5.7)	8,992 (4.9)		
15	5,385 (3.7)	6,227 (3.4)		
16	3,836 (2.7)	4,682 (2.6)		
17	2,907 (2.1)	4,005 (2.2)		
**Residence**			360.302	< 0.001
Urban	79,886 (55.2)	107,038 (58.5)		
Rural	64,822 (44.8)	75,900 (41.5)		
**Digital devices (h/day)**	4.51 ± 2.44	3.79 ± 2.34	85.538	< 0.001
**Physical activity (h/day)**	3.07 ± 0.92	2.85 ± 1.00	65.480	< 0.001

This table reports the N (%) or Mean (SD) of the items.

### Risk factors related to poor visual acuity among children and adolescents

**Figure 1:**
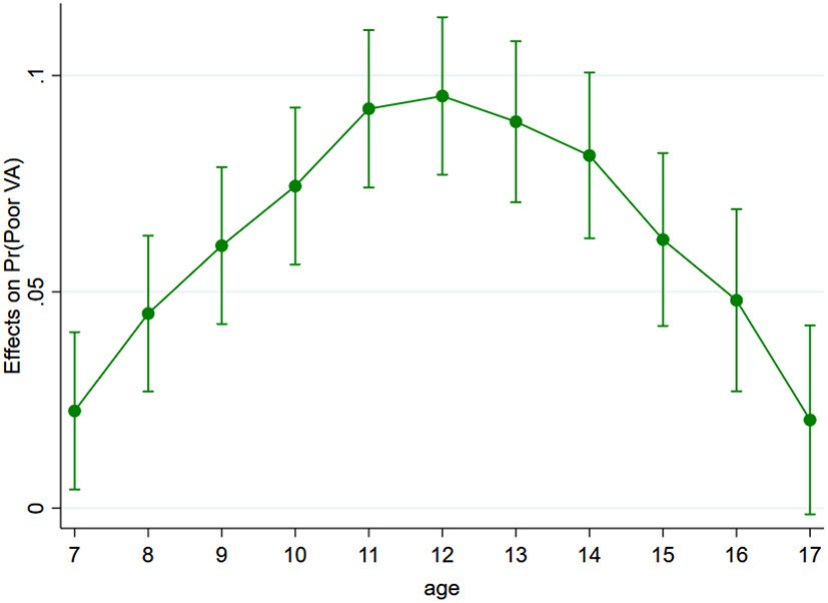
The effects of age on poor visual acuity among children and adolescents.

A logistic regression model was used to analyze the effects of digital device usage and PA on poor visual acuity among children and adolescents during the COVID-19 pandemic. The results showed that the risk factors related to poor visual acuity among children and adolescents included the use of digital devices (OR 1.135; 95% CI 1.132–1.139), and PA (OR 1.269; 95%CI 1.259–1.278). Females had a lower risk of poor visual acuity than males, and rural people had a higher risk than urban ones (Table 2). Age was positively associated with poor visual acuity, and its effect showed an inverted *U*-shape with increasing age (Figure 1).

**Table 2:** Logistic regression model of poor visual acuity among children and adolescents.

**Variables**	***OR***	***SE***	***P***	***95%CI***
**Sex**				
Female	0.913	0.007	< 0.001	0.901–0.926
**Age (year)**				
7	1.102	0.044	0.014	1.020–1.190
8	1.218	0.048	< 0.001	1.128–1.315
9	1.313	0.052	< 0.001	1.216–1.419
10	1.401	0.055	< 0.001	1.297–1.513
11	1.511	0.059	< 0.001	1.398–1.632
12	1.531	0.061	< 0.001	1.417–1.654
13	1.477	0.060	< 0.001	1.365–1.598
14	1.384	0.057	< 0.001	1.276–1.500
15	1.256	0.054	< 0.001	1.155–1.365
16	1.140	0.051	< 0.001	1.045–1.244
17	0.990	0.045	0.828	0.905–1.083
**Residence**				
Rural	1.157	0.008	< 0.001	1.141–1.174
**Physical activity (h/day)**	1.269	0.005	< 0.001	1.259–1.278
**Digital devices (h/day)**	1.135	0.002	< 0.001	1.132–1.139

The time of physical activity and digital devices were calculated as continuous variables.

### The interaction effect of digital devices and age or PA on poor visual acuity

**Figure 2:**
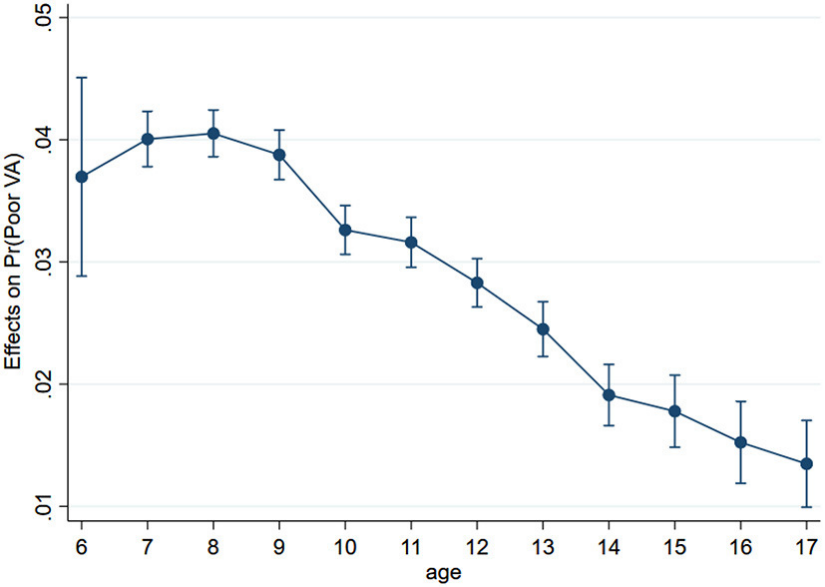
The marginal effects of age and the use time of digital devices on the poor visual acuity among children and adolescents. The results adjusted by sex, residence.

To better understand the effect of digital device usage on poor visual acuity among children and adolescents during the COVID-19 pandemic, we tested whether there was an interaction between age and time spent on digital devices (Table 3). The results showed there are no significant association between the time spent on digital devices and poor visual acuity among children aged 6–11 years (*P* > 0.05). Among adolescents aged 12–17 years, the effects of time spent on digital devices decreased with age (Figure 2). The interaction effects of time spent on PA and digital devices is 0.987. It means that time spent on digital devices is positively associated with poor visual acuity, but the risk of poor visual acuity decreases as the time spent on exercise is increased among children and adolescents.

**Table 3:** The interaction effects of digital devices and age or PA on poor visual acuity.

**Age (years)**	**Digital devices**	***OR*** **(*95%CI*)**	***P***
**Age ×digital devices**			
6 (reference)	3.60 ± 2.01	/	/
7	3.70 ± 1.97	1.011 (0.972–1.052)	0.581
8	3.78 ± 2.08	1.010 (0.972–1.051)	0.603
9	3.95 ± 2.23	1.000 (0.962–1.041)	0.972
10	4.01 ± 2.31	0.973 (0.935–1.011)	0.161
11	4.10 ± 2.39	0.968 (0.931–1.006)	0.097
12	4.34 ± 2.53	0.954 (0.917–0.992)	0.017
13	4.98 ± 2.85	0.938 (0.902–0.976)	0.001
14	5.54 ± 2.99	0.918 (0.882–0.954)	< 0.001
15	5.77 ± 3.04	0.913 (0.877–0.950)	< 0.001
16	6.31 ± 3.10	0.904 (0.868–0.941)	< 0.001
17	6.54 ± 3.17	0.898 (0.862–0.936)	< 0.001
**Digital devices ×PA**	/	0.987 (0.985–0.991)	< 0.001

This table reports the OR and 95%CI of the interaction terms. All models were adjusted by the characteristics listed in Table 2.

## Discussion

The implementation of home quarantine effectively curbed the spread of COVID-19, but it also harmed the mental and physical health of young people. We conducted a study on the poor visual acuity of children and adolescents during the COVID-19 pandemic. The proportion of children and adolescents with poor visual acuity decreased with age. He et al. also found that the proportion of mildly reduced UCDVA among school-aged children and adolescents was relatively higher in primary grades one and two. The proportion of moderately poor visual acuity remained similar among the 12 grades (6–18 years) (27).

We investigated the risk factors of poor visual acuity and found that increased time spent on digital devices due to online courses was the main risk factor for poor visual acuity among children and adolescents. Some studies have indicated that the use of screen devices plays a key role in visual impairment, increasing the possibility of poor visual acuity and myopia (18, 28–30). For instance, a prospective clinical study showed that smartphone use for 4 h resulted in a higher eye disease index than that measured at baseline (31). Liu et al. (32) found that a more myopic spherical equivalent refraction and longer axial length were both associated with more time spent using smartphones and computers but not with time spent using tablets and watching television. In our study, there was a positive association between the use of digital devices and poor visual acuity during the COVID-19 pandemic. We also found that the marginal effect of digital device use on poor visual acuity decreased with age. Similar findings were found by Wang et al. (29) in that the overuse of smartphones was significantly associated with visual impairments, and these visual impairments were more apparent in children than in young adults. This means that the use of digital devices more harmful to younger children than adolescents.

The results of our study suggest that PA is positively correlated with poor visual acuity in children and adolescents. It is contrary to the results of existing studies. Many researchers have found that PA is mildly or not positively associated with myopia and poor visual acuity (33, 34). Some unconventional results must be interpreted carefully. In our study, there was a small difference in the PA time of children and adolescents between cases and controls, ranging from 2.85 to 3.07 h. Some statisticians believe there is a large sample size problem, implying that almost all parameters are significantly different from zero if the sample size is large enough (35–37). Therefore, although we wanted a large sample size to generate more accurate data, an excessively large sample size might cause difficulties interpreting the usual tests of significance (38). This may be the reason for our unusual research results. Meanwhile, we also found the interactions between age and PA are insignificant, but PA can reduce the influence of digital devices on poor visual acuity. Our study is a cross-sectional study rather than a longitudinal cohort study. Therefore, we did not have sufficient evidence to conclude that PA was positively correlated with poor visual acuity, especially this result is contrary to the existing research results.

Some studies have found that time spent outdoors is predictive of the incidence of myopia independent of PA level (15). A systematic review of the correlation between PA and myopia did not find that PA was an independent risk factor for myopia. Instead, the time spent outdoors was identified as the most important factor (34). Dirani et al. (39) suggested that total sports, but not indoor sports, were also significantly negatively associated with myopia. The time spent outdoors had a protective effect on the visual acuity of children and adolescents. However, during the COVID-19 pandemic, children and adolescents were not allowed to play outside. Additionally, the increased use of digital devices was associated with more time at work and less time spent outdoors, resulting in a substitution effect (18, 29, 40). For example, Dirani et al. (40) reported that the lack of adequate outdoor activity might be related to increased time spent on digital devices. However, the substitution effect of the time spent on digital devices and outdoor time is warranted (18, 41, 42). During the COVID-19 pandemic, educational screen time has substituted reading or writing, because of online courses. Thus, poor visual acuity in children and adolescents is associated with home isolation and increased use of digital devices during the COVID-19 pandemic.

Increased use of digital devices was positively associated with poor visual acuity in children and adolescents, and this association decreased with age, but the risk of poor visual acuity decreases as the time spent on PA increases. Parents should strictly control the amount of time students spend on electronics and increase their exercise time, especially outdoor activities. When online courses are necessary, educational institutions should pay more attention to students' eyes use, set reasonable lesson times, and allow students to relax their eyes. Students should ensure that they spend at least 1 h outdoors every day. Parents should focus on the vision of children aged 6 to 11 and help them to develop healthy eye behaviors.

This study has several limitations. Only the duration of PA was measured, and the content and intensity of PA were not included. Therefore, the effect of PA on poor visual acuity could not be accurately determined. We did not collect the frequency and duration of children's daily use of digital devices. However, this was a case-control study with a large sample size of 327,646 children and adolescents. This study partially revealed the negative effects of online courses and the use of digital devices on visual acuity among children and adolescents during the COVID-19 pandemic.

## Conclusion

Our study suggests that children and adolescents were at risk of poor visual acuity during the COVID-19 pandemic. The time spent on digital devices is positively associated with poor visual acuity among children and adolescents during the COVID-19 pandemic, and the association decreased with age. But the risk of poor visual acuity among children and adolescents decreases as the time spent on PA increases. It is essential to pay attention to the negative effect of online courses and home quarantine on visual acuity among children and adolescents.

## Data availability statement

The original contributions presented in the study are included in the article/supplementary material, further inquiries can be directed to the corresponding author/s.

## Ethics statement

The studies involving human participants were reviewed and approved by Ethics Committee of Southern Medical University. Written informed consent to participate in this study was provided by the participants' legal guardian/next of kin.

## Author contributions

CCZ and ZGQ conceived the study. XZ and LS were major contributors in analyzing data and writing the manuscript. WYO, PYL, WH, and YQX mainly collected data. YX, JCZ, and BLX edited and contributed content to the final draft. All authors read and approved the final manuscript.
